# Sample preparation of formalin-fixed paraffin-embedded tissue sections for MALDI-mass spectrometry imaging

**DOI:** 10.1007/s00216-019-02296-x

**Published:** 2020-01-28

**Authors:** Juliane Hermann, Heidi Noels, Wendy Theelen, Michaela Lellig, Setareh Orth-Alampour, Peter Boor, Vera Jankowski, Joachim Jankowski

**Affiliations:** 1 Institute for Molecular Cardiovascular Research, University Hospital RWTH Aachen, Pauwelsstraße 30, 52074 Aachen, Germany; 2 Institute for Pathology, University Hospital RWTH Aachen, Pauwelsstraße 30, 52074 Aachen, Germany; 3 School for Cardiovascular Diseases, Maastricht University, Universiteitssingel 50, 6229 ER Maastricht, The Netherlands

**Keywords:** MALDI imaging, Tissue preparation, Formalin-fixed paraffin-embedded tissue sections, Optimized sample standard operating protocols (SOP)

## Abstract

Matrix-assisted laser desorption/ionization (MALDI) mass spectrometry imaging (MALDI MSI) has become a powerful tool with a high potential relevance for the analysis of biomolecules in tissue samples in the context of diseases like cancer and cardiovascular or cardiorenal diseases. In recent years, significant progress has been made in the technology of MALDI MSI. However, a more systematic optimization of sample preparation would likely achieve an increase in the molecular information derived from MALDI MSI. Therefore, we have employed a systematic approach to develop, establish and validate an optimized “standard operating protocol” (SOP) for sample preparation in MALDI MSI of formalin-fixed paraffin-embedded (FFPE) tissue sample analyses within this study. The optimized parameters regarding the impact on the resulting signal-to-noise (S/N) ratio were as follows: (i) trypsin concentration, solvents, deposition method, and incubation time; (ii) tissue washing procedures and drying processes; and (iii) spray flow rate, number of layers of trypsin deposition, and grid size. The protocol was evaluated on interday variability and its applicability for analyzing the mouse kidney, aorta, and heart FFPE tissue samples. In conclusion, an optimized SOP for MALDI MSI of FFPE tissue sections was developed to generate high sensitivity, to enhance spatial resolution and reproducibility, and to increase its applicability for various tissue types. This optimized SOP will further increase the molecular information content and intensify the use of MSI in future basic research and diagnostic applications.

**Figure:**
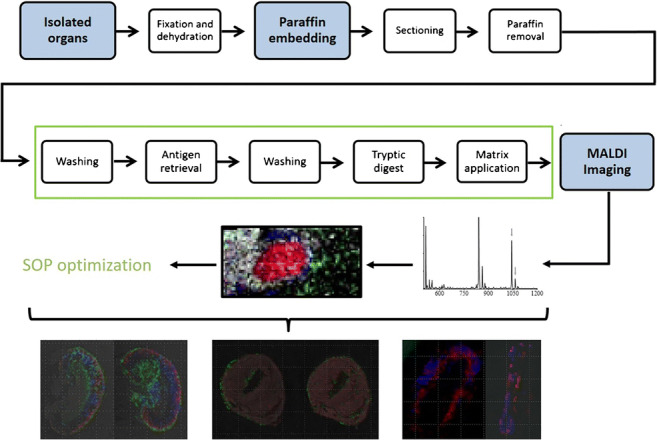
Graphical Abstract

Graphical Abstract

## Introduction

Mass spectrometry has evolved as a powerful bioanalytical tool for identifying and quantifying molecules in biospecimens with high mass accuracy and high sensitivity [1–5]. Matrix-assisted laser desorption/ionization (MALDI) imaging is a variant of mass spectrometry which promises to become of high relevance in histological diagnostics [1, 4, 6–8]. MALDI imaging is more suitable for the analysis of proteins and peptides than other mass spectrometric imaging methods such as DESI (desorption electrospray ionization) or SIMS (secondary ion mass spectrometry). DESI can measure up to 2000 Da, but the resolution is much lower compared with that of MALDI. The mass range of SIMS is limited to 1000 Da. With regard to the combination of a possible mass range up to 100,000 Da and a resolution of up to 20 μm, MALDI is the better choice for protein and peptides. In addition, it is possible to identify the detected signals by MS/MS measurements [9, 10]. MALDI MSI provides resolution information up to 5–10 μm in the spatial distribution and has the ability to identify proteins, peptides, lipids, and small biomolecules in tissue samples. MALDI MSI measurements are performed on the basis of defined geometrical coordinates, covered by an optical image of the tissue, which results in a high accuracy of spatial information on the acquired mass spectra throughout the tissue [11–15]. With this technique, the distribution of more than 100 biomarkers and mediators can be determined in a single tissue analysis by multi-channel detection [6].

Sample preparation for MALDI MSI measurements includes tissue sectioning, enzymatic digestion, matrix coating, and determination of experimental conditions that ensure optimal ionization of the sample. Optimization of these steps while ensuring the reproducibility of the results is crucial to obtaining high-quality MALDI MSI data [1, 11–14, 16]. However, even minor changes in the procedure for slide preparation of tissues could induce significant changes in the accuracy, sensitivity, and reproducibility of MALDI MSI data (e.g. [15]).

MALDI MSI is generally used for the analysis of fresh-frozen tissue sections and biopsies of various origins (e.g. liver, aorta, kidney, brain), and has been successfully applied in both preclinical and clinical research focussing on biomarkers and mediators of (patho-)physiological processes in various applications [6–8, 17–20] and diagnostic approaches. Thus, many different protocols are available for the analysis of fresh-frozen sections (e.g. [7, 21, 22]) and for diagnostic application (e.g. [23, 24]).

However, MALDI MSI techniques are not available in all (patho-)histological centres. Therefore, shipment and procurement of fresh tissue specimens without degradation is essential. But, as demonstrated in various studies, this extremely challenging constraint has hindered endeavours to successfully guarantee the required robustness of the analyses [11, 25–27]. Formalin-fixed paraffin-embedded (FFPE) tissue sections offer an interesting alternative to overcome this limitation. FFPE samples have very limited tissue degradation processes, are routinely used in both clinical diagnostics and research, and can be stored at room temperature.

The removal of paraffin represents a critical obstacle, as this step implies the risk of modifying or removing endogenous components such as proteins, peptides, lipids, or low-molecular-weight substances within the tissue samples along with the desired paraffin removal. Therefore, efficient sample preparation is essential to the collection of valid data from these tissue sections. Consequently, MALDI MSI measurement of FFPE sections, including proteomics analyses, is vitally needed [28, 29]. Diehl et al. described enzymatic digestion of FFPE sections rendering mass spectrum results comparable with the analysis of fresh-frozen tissue samples [15]. Protocols for the analysis of protein in FFPE tissue samples have also been described, e.g. by Casadonte et al. [30] as well as by O’Rourke [31, 32]. These protocols are, generally speaking, in part comparable, while some details differ considerably, making evident the necessity for the identification of the best combination of steps for sample preparation to enable robust MALDI MSI of fixed biospecimens. Therefore, in this study, we have developed, established, optimized, and validated a “standard operating protocol” (SOP) for the preparation of FFPE sections for MALDI MSI for research approaches and clinical diagnostics.

## Materials and methods

Ethanol, 2,5-dihydroxybenzoic acid, polylysine, paraformaldehyde (PFA), trifluoroacetic acid (TFA), and chloroform were purchased from Sigma-Aldrich (Taufkirchen, Germany). Acetic acid and citric acid were purchased from Merck (Darmstadt, Germany) and n-isopropanol and hydroxymethyl-aminomethane hydrochloride from Roth (Karlsruhe, Germany). Ammonium bicarbonate was obtained from Riedel de Haen (Seelze, Germany) and ultrapure xylene from Otto Fischar (Merchweiler, Germany). Liquid chromatography (LC)-MS–grade acetonitrile was purchased from Fisher Chemicals (Zurich, Switzerland), and high-pressure liquid chromatography (HPLC)–grade water was generated with a “Milli-Q Advantage A10” apparatus from Merck (Darmstadt, Germany).

### Tissue sample preparation

After sacrificing the mice, the organs (kidney, heart, and aorta) were rinsed with 4% paraformaldehyde (PFA) solution before being isolated and fixed in PFA at 4 °C overnight. Next, the tissues were dehydrated, using an MTM automated tissue processor (SLEE Medical GmbH, Mainz, Germany), and embedded in paraffin before sectioning, using a “microtome” (Leica RM 2245, Wetzlar, Germany). The thickness of the tissue sections was fixed at 5 μm. The sections were mounted on indium tin oxide (ITO) conductive–coated slides (Hudson Surface Technology, New York, USA). Before mounting, the tissue sections were additionally coated with polylysine [33]. In addition, fresh-frozen tissue sections were prepared from mouse kidneys by rinsing the organs with 4% paraformaldehyde (PFA) solution before isolation and fixing in PFA at 4 °C overnight.

### Optimization of sample preparation for MALDI MSI

Prior to MALDI MSI analysis, tissue sections were rehydrated and deparaffinized by washing twice with xylene for 2 min each followed by washes in a series of isopropanol aqueous solutions of decreasing concentrations for 3 min each, as described previously [34]. Afterwards, tissue sections were incubated in 10-mM citric acid solution at 85 °C using an incubator (Heraeus, Hanau, Germany) for 60 min, followed by incubation for 20 min in 10 mM citrate acid at room temperature for antigen retrieval. Glass slides were dipped in aqueous 20 mM ammonium bicarbonate for 5 min at room temperature and vacuum-dried for 15 min. Trypsin was deposited onto the tissue sections in 25 layers using a 50-nM aqueous trypsin solution, dissolved in aqueous 20 mM ammonium bicarbonate at a spray flow rate of 10 μl/min at room temperature. The solution was sprayed using a MALDI spotter at a speed of 2.6 s/cm^2^ and an air flow of 0.4–0.5 l/min using an air pressure of 1 bar (SunChrom, Friedrichsdorf, Germany). Hereby, the aqueous trypsin solution was applied to the tissue sections as a spray mist. After drying at room temperature, matrix deposition for MALDI analysis was performed seven times using a solution of 0.1 mM 2,5-dihydroxybenzoic acid, dissolved in 0.1% TFA with 10% acetonitrile at a spray flow rate of 7 μl/min. The spray conditions were as described above. The matrix solution was subsequently dried at room temperature.

### Optimization of the trypsin solution and digestion period

First, the effect of trypsinization of the tissue sections on the S/N of the acquired mass spectrometric data was tested using trypsin (50 nM in 20 mM aqueous ammonium bicarbonate) from different manufacturers ((a) Sigma-Aldrich, product number: T6567; (b) Promega, product number: V5117; (c) Roche, product number: 11418025001). Second, the solvent for dissolving trypsin (50 nM) was modified to maximize the S/N ratio of the MALDI MSI measurement. For this, the effect of water, 20 mM ammonium bicarbonate, 20 mM ammonium bicarbonate with 10% acetonitrile, and 50 mM hydroxymethyl-aminomethane hydrochloride at pH 8.3 was analyzed [15]. Third, the effect of increased concentrations of trypsin (5 and 50 nM) on the S/N ratio of the resulting MALDI MSI spectra was analyzed. To maximize the number of fragments of enzymatically digested proteins in the tissue sections, and thus the sensitivity, three approaches were analyzed: trypsin deposition (i) without incubation period, (ii) with an incubation period of 120 min, and (iii) with an incubation period of 24 h, each at 37 °C in a humidity chamber [15, 35]. After identifying ammonium bicarbonate as the optimal solvent for trypsin, the concentration of the ammonium bicarbonate solution was further optimized to increase the resulting S/N ratio.

Next, the effect of the drying time between the single layers of trypsin deposition on the S/N ratio of the resulting MALDI MSI spectra was investigated. The aqueous trypsin solution was sprayed without and with a drying time of 5 min to ensure proper drying of the coating [36]. Also, the method for drying the tissue sections just prior to trypsin deposition was further optimized: the tissue sections were dried at room temperature as well as with a vacuum desiccator for 15 min, respectively, as suggested by several working groups [21, 22, 28, 29, 37].

Further, as proposed by Gustafsson et al. [38], it was examined whether an additional washing step of the tissue sections using 20 mM ammonium bicarbonate in water after the antigen retrieval and before the enzymatic digestion, further increases the S/N ratio of the MALDI MSI data.

### Effect of differential washing steps before and after antigen retrieval

The washing process of the tissue sections after paraffin removal was further optimized by analyzing combinations of different washing procedures. Two washing protocols were used, namely the “basic wash” and “Carnoy’s wash” as described in Kelley et al. [39]. Briefly, for the “basic wash”, the deparaffinized tissue sections were washed subsequently in 70% isopropanol, 100% isopropanol, and pure water for 2 min each. For “Carnoy’s wash” procedure, the removal of paraffin by xylene was followed by washing with 70% and 100% ethanol, Carnoy’s solution (ethanol-chloroform-acetic acid in 6:3:1 parts, respectively), 100% ethanol, 0.1% TFA, and 100% ethanol for 30 s each [11]. The different combinations of the various washing processes analyzed in this optimization study are shown in Table 1.

**Table 1:** Overview of the optimized steps for the establishment of an optimized SOP for the preparation of FFPE sections for MALDI MSI

Optimized parameters	Optimal	
Optimization of trypsin solution and digestion period		
Step 1: trypsin supplier	*Sigma-Aldrich*	*x*
	Promega	
	Roche	
Step 2: trypsin solvent	Water	
	*20 mM ammonium bicarbonate*	*x*
	20 mM ammonium bicarbonate + 10% acetonitrile	
	50 mM hydroxymethyl-aminomethane hydrochloride at pH 8.3	
Step 3: trypsin concentration	5 nM	
	*50 nM*	*x*
Step 4: additional incubation time after application of trypsin solution (35 min)	*0 min*	*x*
	120 min	
	24 h	
Step 5: concentration of ammonium bicarbonate as trypsin solvent	*20 mM*	*x*
	50 mM	
	80 mM	
Step 6: trypsin drying time between trypsin coating steps	*0 min*	*Comparable*
	*5 min*	*Comparable*
Step 7: tissue drying method before trypsin deposition	*Room temperature 15 min*	*Comparable*
	*Vacuum desiccator 15 min*	*Comparable*
Step 8: wash in 20 mM ammonium bicarbonate after antigen retrieval	*With additional wash*	*Comparable*
	*Without additional wash*	*Comparable*
Optimization of washing steps before and after antigen retrieval		
Step 9: washing steps before and after antigen retrieval (*for details, see Table 1 in main text)	1) Xylene, basic wash, antigen retrieval	
	2) Xylene, basic wash, antigen retrieval, basic wash	
	3) Xylene, basic wash, antigen retrieval, Carnoy’s wash	
	4) *Xylene, Carnoy’s wash, antigen retrieval*	*x*
	5) Xylene, Carnoy’s wash, antigen retrieval, basic wash	
	6) Xylene, Carnoy’s wash, antigen retrieval, Carnoy’s wash	
Optimization of spray flow rate, number of layers of trypsin deposition, and grid size		
Step 10: spray flow rate	2 μl/min	
	*5 μl/min*	*x*
	10 μl/min	
	15 μl/min	
Step 11: number of layers trypsin deposition	5	
	10	
	*15*	*x*
	20	
Step 12: grid size for MALDI-MS imaging	*20 μm*	*x*
	50 μm	
	100 μm	

### Effect of the spray flow rate, number of layers of trypsin deposition, and grid size

For trypsin and matrix deposition, a MALDI spotter should be used to ensure homogeneous trypsin and matrix deposition. The effect of increased spray flow rates (2 μl/min, 5 μl/min, 10 μl/min, and 15 μl/min) and number of deposited trypsin layers (5, 10, 15, and 20) were optimized to the resulting MALDI MSI data [8]. Finally, the effect of decreasing grid sizes (100 μm, 50 μm, and 20 μm) was analyzed.

### Mass spectrometric conditions and data analysis

MALDI MSI was performed using a MALDI Ultraflex III TOF/TOF mass spectrometer (Bruker Daltonics, Bremen, Germany) equipped with a 337-nm N_2_ laser with a 66.7-Hz repetition rate and controlled by Flex-Control 3.3. With a laser spot size of 10 μm, we used a distance of 50 μm between the spots, unless otherwise stated in the paper. MS spectra were acquired with 100 laser shots per position in reflector-positive mode. The ubiquitous applicability of the SOP was validated by using a Rapiflex mass spectrometer controlled by the Flex-Control 4.0 (Bruker Daltonics, Bremen, Germany). MS spectra were acquired with 800 laser shots per position in reflector-positive mode. Analyses were taken in mass range 600–1500 Da. To measure the organs with a high resolution, a grid size of 10 μm was used. Data were analyzed with the Flex-Imaging 2.1 software (Bruker Daltonics, Bremen, Germany), and MALDI MSI data were normalized using the “root mean square” (RMS) [40, 41]. To analyze the impact of the modification of a protocol parameter on the sensitivity and reproducibility of the MALDI MSI measurement, the signal-to-noise (S/N) ratio was calculated for specific mass signals using the SCiLS software (Ver. 2016b). Briefly, the S/N was calculated by dividing the difference between the peak intensity and median by the median absolute deviation (MAD). Using median and MAD guarantees a robust estimation and compatibility for S/N calculation. Different mass signals were chosen for illustration of MALDI MSI images that present specific localizations in the tissue. The identification of peptides was performed through the MS/MS spectra using the lift option of the MALDI Ultraflex III TOF/TOF mass spectrometer (Bruker Daltonics, Bremen, Germany). The mass spectra were calibrated and annotated using BioTools 3.0 (Bruker Daltonics, Bremen, Germany) in combination with the MASCOT 2.2 database (Matrix Science, London, UK) comparing experimental mass spectrometric data with calculated peptide masses for each entry into the sequence database. Analysis with a genomic database allowed the amino acid sequence to be determined and assigned to a protein.

### Statistical analyses

All statistical analyses were performed using GraphPad Prism Version 6 (GraphPad Software Inc.). S/N ratios of MALDI MSI analyses are shown as means ± SEM. After testing for normality, the unpaired one-tailed *t* test was used to compare the S/N ratios of mass signals obtained using different protocols. Interday variability was calculated based on the total ion count of consecutive tissue slide mass spectrometric analyses on three consecutive days. A *P* < 0.05 was considered statistically significant.

## Results

**Fig. 1:**
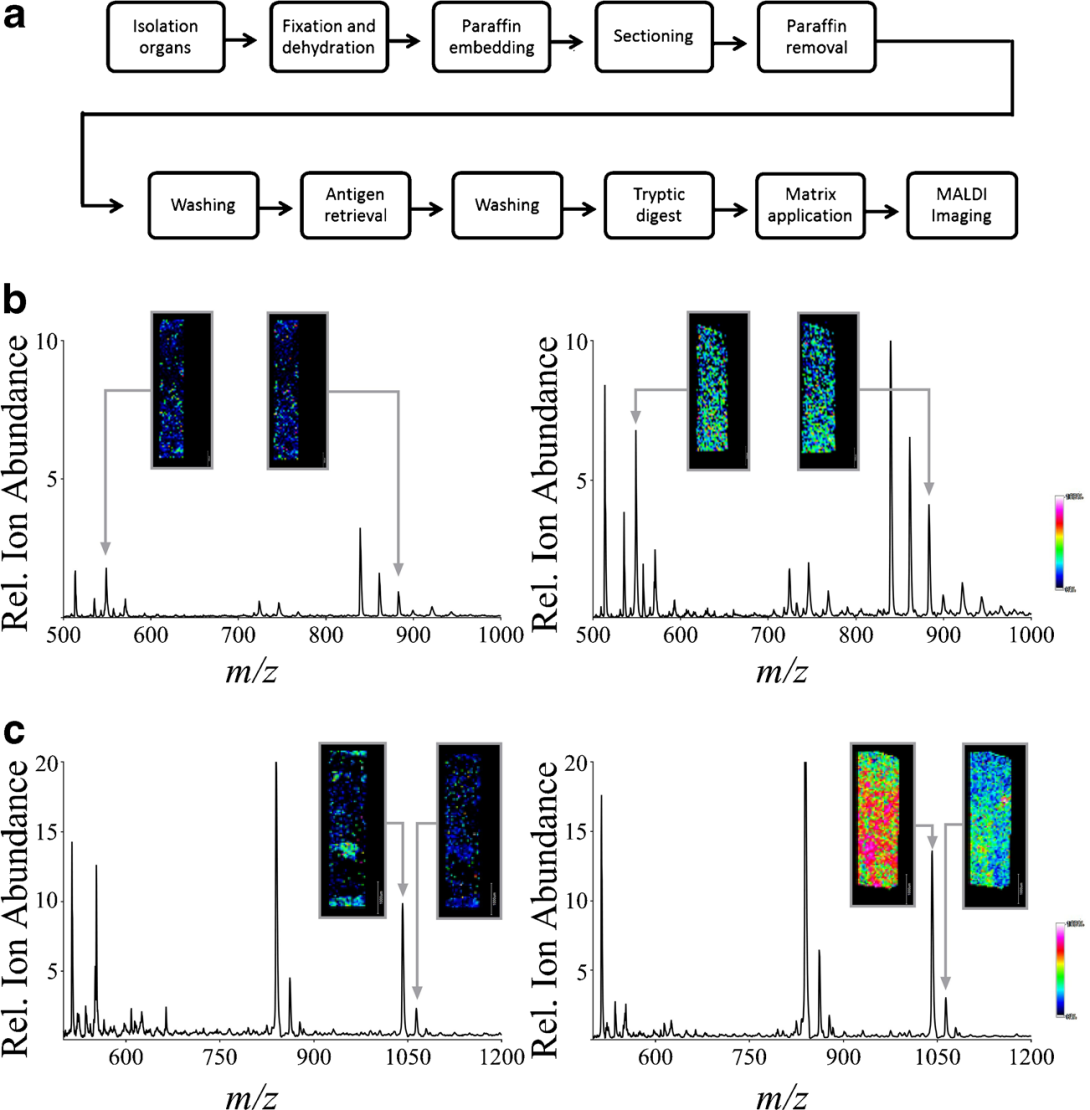
Optimizing an SOP for the MALDI MSI analysis of FFPE samples and the effect of trypsin manufacturers and solvents on MALDI MSI data. **a** Schematic representation of process steps optimized for MALDI MSI imaging of FFPE samples. **b** The effect of solvents for dissolving trypsin on MALDI MSI spectra. Shown are characteristic MALDI MSI spectra of mouse kidney samples when using 50 mM hydroxymethyl-aminomethane hydrochloride (left) vs. 20 mM ammonium bicarbonate (right) to dissolve trypsin from Sigma-Aldrich. **c** The effect of increasing concentrations of trypsin for enzymatic digestion of tissue proteins. Shown are characteristic MALDI MSI spectra using a trypsin concentration of 5 nM (left) vs. 50 nM (right). *^)^Grid size 50 μm each

Figure 1a illustrates the overview of the development of the “standard operating protocol” (SOP) (Table 1). In detail, the following parameters were varied to reach the aim of an optimized SOP:

### Effect of the trypsin solution and digestion period

Initially, trypsin preparations purchased from different manufacturers were screened to achieve a maximal S/N ratio of MALDI MSI data. Trypsin from each manufacturer was dissolved in 20 mM ammonium bicarbonate with a trypsin concentration of 50 nM. This was achieved based on the randomly selected base mass signal of the tissue signal 840 ± 0.3 (*m*/*z*), whose amino acid sequence belongs to CCARYPK, which may originate from the Cx9C motif-containing protein 4. The peptide achieved a maximal S/N ratio using trypsin purchased from Sigma-Aldrich (*trypsin from Sigma-Aldrich* vs. *trypsin from Roche 0.278 ± 0.022* vs. *0.067 ± 0.006* (*P < 0.05*); *trypsin from Sigma-Aldrich* vs. *trypsin from Promega 0.278 ± 0.006* vs. *0.098 ± 0.023* (*P < 0.05*); *n = 3*). The S/N was used to optimize the experimental conditions, as other parameters, such as number of identifiable peptides, were found to be unsuited for optimization due to the heterogeneity of kidney and heart tissues.

The effects of different solvents for trypsin on MALDI MSI mass signal intensities are demonstrated in Fig. 1b. The data given in the figure are representative spectra from single points of the tissue sample. Figure 1b (left panel) shows a spectrum from a mouse kidney sample taken after incubation with trypsin (50 nM) from Sigma-Aldrich dissolved in hydroxymethyl-aminomethane hydrochloride (50 mM); Fig. 1b (right panel) shows the resulting MALDI MSI spectrum using 20 mM ammonium bicarbonate to dissolve trypsin (50 nM) from Sigma-Aldrich. The use of hydroxymethyl-aminomethane hydrochloride resulted in spectra with the minimum S/N ratio of all tested solvents; the use of ammonium bicarbonate resulted in the maximum S/N ratio. This was achieved based on the randomly selected base mass signals of the tissue signal 549 ± 0.3 (*m*/*z*), whose amino acid sequence belongs to EASDK, which may originate from metallothionein-2, and 882 ± 0.3 (*m*/*z*), whose amino acid sequence belongs to CCLKYPR, which may originate from beta-defensin 29. The peptides achieved a maximal S/N ratio using 20 mM ammonium bicarbonate (*50 mM hydroxymethyl-aminomethane hydrochloride* vs. *20 mM ammonium bicarbonate*: *for 549* (*m/z*) *0.053 ± 0.011* vs. *0.230 ± 0.007* (*P < 0.05*); *for 882* (*m/z*) *0.068 ± 0.009* vs. *0.167 ± 0.025* (*P < 0.05*); *H*_*2*_*O* vs. *20 mM ammonium bicarbonate: for 549* (*m/z*) *0.064 ± 0.017* vs. *0.230 ± 0.007* (*P < 0.05*); *for 882* (*m/z*) *0.076 ± 0.007* vs. *0.167 ± 0.025* (*P < 0.05*); *20 mM ammonium bicarbonate + 10% acetonitrile* vs. *20 mM ammonium bicarbonate: for 549* (*m/z*) *0.072 ± 0.008* vs. *0.230 ± 0.007* (*P < 0.05*); *for 882* (*m/z*) *0.076 ± 0.006* vs. *0.167 ± 0.025* (*P < 0.05*); *n = 3*).

The effects of increasing concentrations of trypsin for enzymatic digestion of tissue proteins were subsequently analyzed. Figure 1c (left panel) shows a characteristic MALDI MSI spectrum using 5 nM trypsin in a 20-mM ammonium bicarbonate solution. By using a 50-nM trypsin solution (Fig. 1c (right panel)), the S/N ratios significantly increased. The maximum S/N ratios of the selected mass signals (*1041 m/z and 1063 m/z*) 1041 ± 0.3 (*m*/*z*), whose amino acid sequence belongs to KVPGFCSFR, which may originate from the sodium-dependent phosphate transport protein 1, and 1063 ± 0.3 (*m*/*z*), whose amino acid sequence belongs to NGRENVCSGK, which may originate from the C-C motif chemokine in the MALDI MSI spectra, were achieved by using a trypsin concentration of 50 nM (*50 nM trypsin* vs. *5 nM trypsin*: *for 1041* (*m/z*) *1.151 ± 0.078* vs. *0.069 ± 0.016* (*P < 0.05*); *for 1063* (*m/z*) *2.150 ± 0.194* vs. *0.0830 ± 0.018* (*P < 0.05*); *n = 3*).

**Fig. 2:**
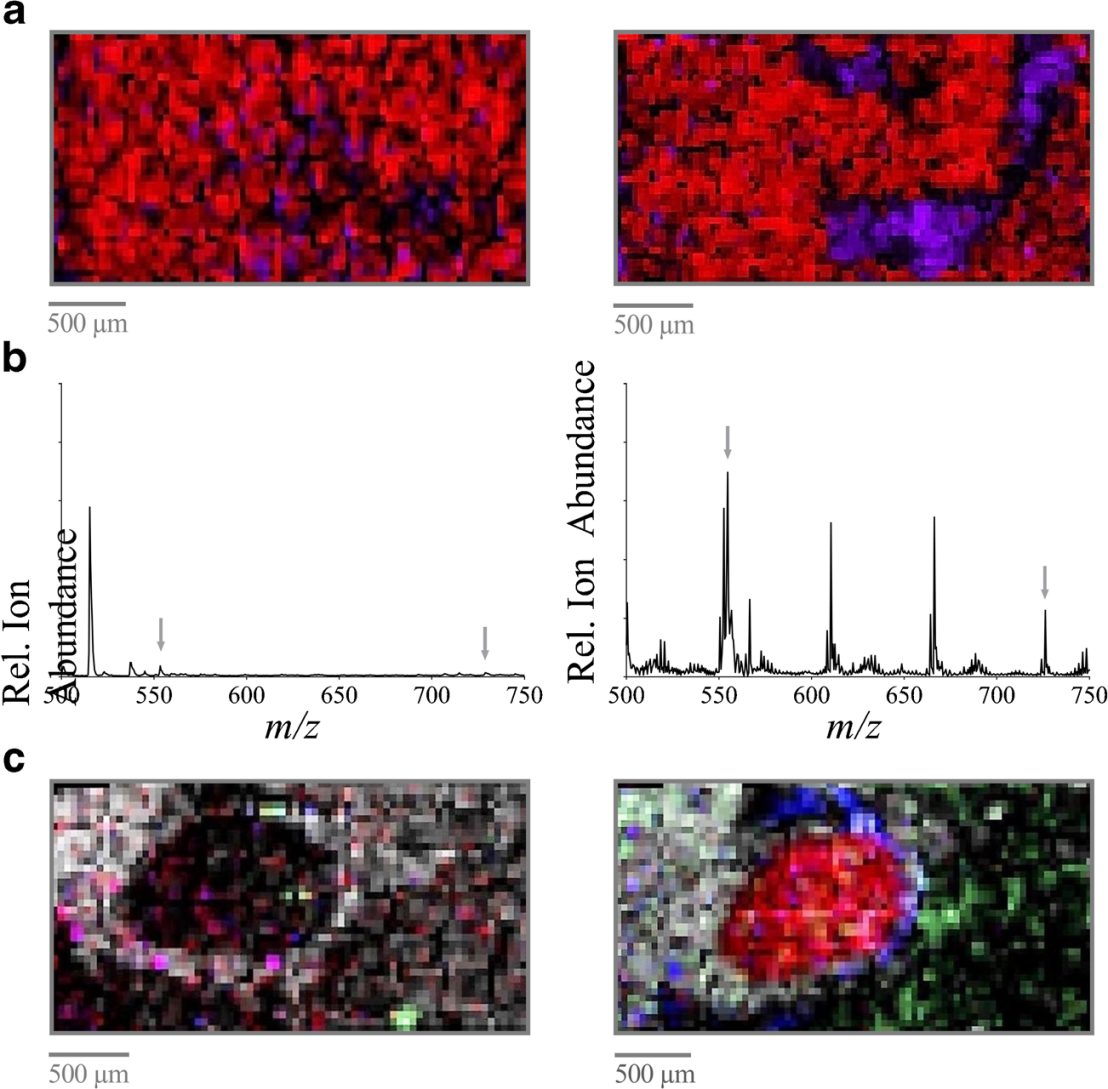
The effect of trypsin solutions, incubation times, and coating procedures on MALDI MSI data. **a** The effect of the duration of the trypsin incubation period. Shown are MALDI MSI images of the mass signal at 680 (*m/z*) purple and 843 (*m/z*) red in a kidney section after trypsin incubation for 120 min at 37 °C (left) vs. without an additional incubation period after trypsin spraying (35 min) (right). **b** The effect of varying concentrations of ammonium bicarbonate for dissolving trypsin. Shown is a characteristic single point MALDI MSI spectrum using 80 nM (left) vs. 20 nM (right) ammonium bicarbonate concentration for dissolving the trypsin. **c** The effect of a 5-min delay between coating layers of trypsin. Shown are MALDI MSI images of the mass signals at 907 (*m/z*) grey, 1712 (*m/z*) blue, 1920 (*m/z*) red, and 2751 (*m/z*) green in a kidney section on which trypsin layers were coated with a 5-min delay between the single coating layers (left) vs. a continuous trypsin deposition without any delay during the spray process (right). *^)^Grid size 50 μm each

Next, the effect of the duration of the incubation period of trypsin on MALDI MSI mass signals was analyzed. Exemplary images of the mass spectrometric data with the minimum and maximum S/N ratio are presented. Figure 2a (left panel) shows characteristic MALDI MSI data of a kidney section accumulated after a trypsin incubation period of 120 min at 37 °C as suggested by Diehl et al. and De Sio et al. [15, 42]. Figure 2a (right panel) shows the MALDI MSI data from the tissue section without an additional incubation period after trypsin spraying. Improved results in terms of quality of the resulting images were achieved without additional trypsin incubation time, as demonstrated by a higher signal intensity as well as lower signal scatter inside a rather homogeneous region (right panel) for the selected mass signals 680 ± 0.3 (*m*/*z*), whose amino acid sequence belongs to GVIQHK, which may originate from the ubiquitin-fold modifier-conjugating enzyme 1, and 843 ± 0.3 (*m*/*z*), whose amino acid sequence belongs to CVPPHYK, which may originate from the mitochondrial import inner membrane translocase subunit 10. A further increase in the incubation time to 24 h did not result in increased signal intensities (*no incubation* vs. *120*-*min incubation: for 680* (*m/z*) *1.920 ± 0.088* vs. *0.095 ± 0.023* (*P < 0.05*); *for 843* (*m/z*) *0.139 ± 0.008* vs. *0.091 ± 0.025* (*n*.*s*.); *no incubation* vs. *24*-*h incubation: for 680* (*m/z*) *1.920 ± 0.088* vs. *0.112 ± 0.012* (*P < 0.05*); *for 843* (*m/z*) *0.139 ± 0.008* vs. *0.0972 ± 0.008* (*P < 0.05*); *n = 3*).

The effects of varying concentrations (80 mM, 50 mM, 20 mM) of ammonium bicarbonate for dissolving 50 nM trypsin on the resulting MALDI MSI mass signals were also investigated. Exemplary spectra of the mass spectrometric data with the minimal S/N ratio and maximal S/N ratio are presented. A characteristic single point MALDI MSI spectrum, as shown in Fig. 2b (left panel), demonstrates the effect of using an 80-mM ammonium bicarbonate concentration to dissolve trypsin. In contrast, Fig. 2b (right panel) shows the corresponding MALDI MSI spectrum when using a concentration of 20 mM ammonium bicarbonate. The maximum S/N ratios of the mass signals with the maximum intensities in the MALDI MSI spectra 552 ± 0.3 (*m*/*z*), whose amino acid sequence belongs to TLYR, which may originate from the 28S ribosomal protein S10, and 726 ± 0.3 (*m*/*z*), whose amino acid-sequence belongs to NAPVGIR, which may originate from the mitochondrial import inner membrane translocase subunit 17, were achieved using 20 mM ammonium bicarbonate to dissolve trypsin (*80 mM* vs. *20 mM: for 552* (*m/z*) *0.068 ± 0.009* vs. *0.269 ± 0.019* (*P < 0.05*); *for 726* (*m/z*) *0.072 ± 0.006* vs. *0.246 ± 0.060* (*P < 0.05*); *50 mM* vs. *20 mM: for 552* (*m/z*) *0.080 ± 0.004* vs. *0.269 ± 0.019* (*P < 0.05*); *for 726* (*m/z*) *0.080 ± 0.007* vs. *0.246 ± 0.060* (*P < 0.05*); *n = 3*).

Next, the effect of a delay while coating the samples with trypsin on the resulting MALDI MSI imaging was analyzed, as depicted in Fig. 2c. Figure 2c (left panel) shows a MALDI MSI image of a tissue section on which trypsin layers were coated with a 5-min delay between the individual coating layers. Different mass signals were chosen to analyze specific regions of the tissue. The MALDI MSI image in Fig. 2c (right panel) demonstrates the effect of a continuous trypsin deposition without a delay in the spray process of trypsin layers. A delay of 5 min had no significant effect on S/N ratios of the resulting MALDI MSI data as exemplary calculated for the *m*/*z* signals at 907 ± 0.3 (*m*/*z*), whose amino acid sequence belongs to QLADIGYK, which may originate from the cytochrome c oxidase assembly protein COX 14; 1712 ± 0.3 (*m*/*z*), whose amino acid sequence belongs to VLVVGDAAVGKTSLVQR, which may originate from the Ras-related protein Rab-7L1; and 2751 ± 0.3 (*m*/*z*), whose amino acid sequence belongs to FPFCDGAHIKHNEETGDNCGPLIIK, which may originate from the CDGSH iron-sulfur domain-containing protein 1, respectively (with vs. without delay during spray process: *907* (*m/z*) *0.223 ± 0.011* vs. *0.129 ± 1.260* (*n.s.*); *for 1712* (*m/z*) *0.821 ± 0.156* vs. *1.428 ± 0.450* (*n.s.*); *for 2751* (*m/z*) *0.190 ± 0.013* vs. *0.125 ± 0.420* (*n.s.*); *n = 3*).

An additional washing step with 20 mM ammonium bicarbonate after deparaffinization and antigen retrieval for 2 min did not result in a further increase in signal sensitivity on MALDI MSI mass signals nor did it result in an increase in intensity (*additional washing step with 20 mM ammonium bicarbonate* vs. *no additional washing step: for 918* ± 0.3 (*m*/*z*), whose amino acid sequence belongs to FHSQLM, which may originate from the signal recognition particle 9 protein *0.236 ± 0.068* vs. *0.374 ± 0.070* (*n*.*s*.); *n = 3*).

Finally, the effect of drying tissue sections using a vacuum desiccator at room temperature just before trypsin deposition was analyzed. However, no significant differences were detected in the resulting MALDI MSI data (*air drying* vs. *vacuum*: *for* 660 ± 0.3 (*m*/*z*), whose amino acid sequence belongs to GLENID, which may originate from the NADH dehydrogenase-ubiquinone-1 alpha subcomplex subunit-1; *1.042 ± 0.064* vs. *0.895 ± 0.312* (*n*.*s*.); *n = 3*).

### Effect of different tissue washing procedures before and after antigen retrieval

**Fig. 3:**
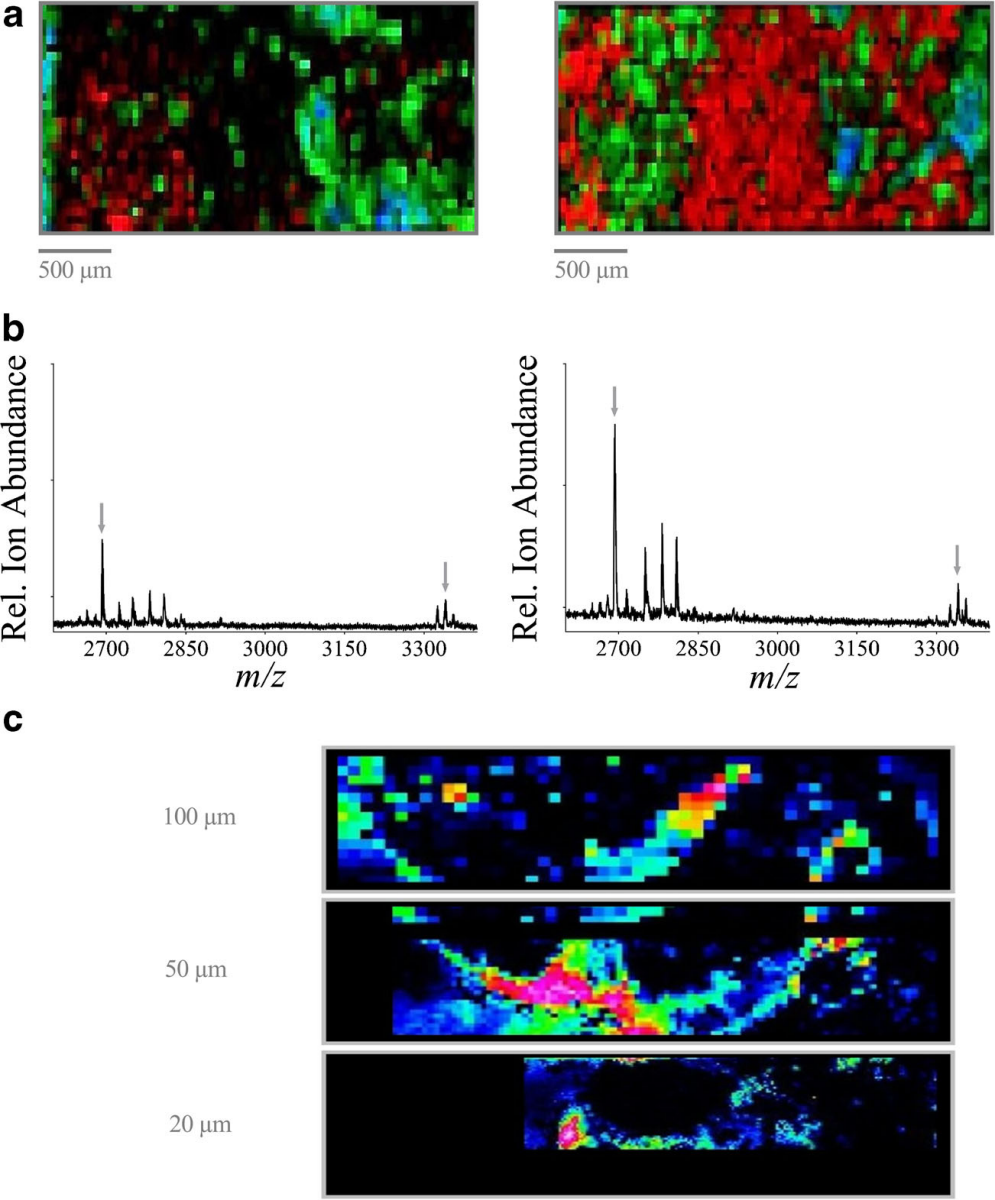
The effect of tissue washing procedures, trypsin coating spray flow rates, and grid sizes on MALDI MSI data. **a** The effect of tissue washing procedures before trypsin coating. Shown are MALDI MSI images of the mass signals at 904 (*m/z*) blue, 1215 (*m/z*) red, and 2809 (*m/z*) green in a kidney section subjected to a “basic wash” procedure (left) vs. “Carnoy’s wash” (right) before antigen retrieval and trypsin coating. *Grid size 50 μm. **b** The effect of trypsin coating spray flow rates. Shown are MALDI MSI spectra using a spray flow rate of 15 μl/min (left) vs. 5 μl/min (right). *Grid size 50 μm. **c** The effect of grid size. Shown are MALDI MSI images of the mass signals at 237 (*m/z*) with intensity distribution in a kidney section imaged using grid sizes of 100 μm, 50 μm, and 20 μm, as indicated

To investigate the effect of a specific tissue washing procedure on the tissue sections before trypsin coating on MALDI MSI mass signals, two protocols for washing the tissue sections were analyzed in different combinations, both before and after antigen retrieval (Table 1). Exemplary images of the mass spectrometric data with the minimum S/N ratio and with the maximum S/N ratio are presented. Further, different mass signals were chosen to analyze specific regions of the tissue. The MALDI MSI data given in Fig. 3a (left panel) demonstrate the effect of a washing procedure that basically removes xylene and induces the rehydration of the tissue after xylene-mediated paraffin removal by washing in alcoholic solutions (“basic wash” procedure) before antigen retrieval. In comparison, the effect of two additional washing solutions, Carnoy’s solution and 0.1% TFA in Carnoy’s washing procedure before antigen retrieval (Table 1; step 9 approach 4), is demonstrated in Fig. 3a (right panel). Of all washing approaches analyzed, the optimal results were reached using xylene, followed by Carnoy’s wash protocol and antigen retrieval, as displayed by an overall higher signal intensity and lower signal scatter inside rather homogeneous regions (right panel) for selected mass signals. The S/N ratios of the base signals in the resulting MALDI MSI spectra (904 ± 0.3 (*m*/*z*), whose amino acid sequence belongs to FGVAEPRK which may originate from the cytochrome c oxidase subunit 6C; *1215* ± 0.3 (*m*/*z*), whose amino acid sequence belongs to IPSTDANPAGGGK, which may originate from the Ras-related protein Rab-22A; and 2809 ± 0.3 (*m*/*z*), whose amino acid sequence belongs to AAGPQSSGAAVSAAAYPDSPVELPARLQK, which may originate from the transmembrane protein 42) were significantly increased or comparable when using Carnoy’s wash protocol compared with those when using all other protocols in Table 1 (*basic* vs. *Carnoy’s*: *for 904* (*m/z*) *0.081 ± 0.011* vs. *0.276 ± 0.189* (*P < 0.05*); *for 1215* (*m/z*): *0.094 ± 0.007* vs. *0.196 ± 0.015* (*P < 0.05*); *for 2809* (*m/z*) *0.094 ± 0.004* vs. *0.121 ± 0.148* (n.s.)).

### Effect of the spray flow rate and number of layers of trypsin deposition

Next, the spray flow rates (2 μl/min, 5 μl/min, 10 μl/min, and 15 μl/min), performed using a MALDI spotter (Micro-Fraction Collector; SunChrom), applied for trypsin deposition, were optimized to the achieved S/N ratio. Trypsin was nebulized using nitrogen and sprayed to the tissue sample. Exemplary spectra of the mass spectrometric data with the minimum and with the maximum S/N ratio, respectively, are presented. A spray flow rate of 15 μl/min results in MALDI MSI data as exemplified in Fig. 3b (left panel). Figure 3b (right panel) presents MALDI MSI data using a spray flow rate of 5 μl/min for trypsin deposition. The maximum S/N ratios of the resulting mass signals with the highest intensity at 2694 ± 0.3 (*m*/*z*), whose amino acid sequence belongs to DPLRAQQLAAELEVEMMADMYNR, which may originate from the mitochondrial import inner membrane translocase subunit 10, and 3340 ± 0.3 (*m*/*z*), whose amino acid sequence belongs to FLAFTCGLLCGALHTLGFQSLVTASVASLPACK, which may originate from the trafficking protein particle complex subunit 6A, were achieved using a spray flow rate of 5 μl/min (*15 μl/min* vs. *5 μl/min: for 2694* (*m/z*) *0.103 ± 0.002* vs. *0.515 ± 0.207* (*P < 0.05*); *for 3340* (*m/z*) *0.097 ± 0.005* vs. *0.250 ± 0.068* (*P < 0.05*); *2 μl/min* vs. *5 μl/min: for 2694* (*m/z*) *0.097 ± 0.003* vs. *0.515 ± 0.207* (*P < 0.05*); *for 3340* (*m/z*) *0.096 ± 0.004* vs. *0.250 ± 0.068* (*P < 0.05*); *10 μl/min* vs. *5 μl/min*: *for 2694* (*m/z*) *0.107 ± 0.005* vs. *0.515 ± 0.207* (*P < 0.05*); *for 3340* (*m/z*) *0.108 ± 0.010* vs. *0.250 ± 0.068* (*n*.*s*.); *n = 3*).

Subsequently, the effect of an increased number of trypsin layers on the resulting MALDI MSI data was analyzed. The maximum S/N ratio for the MALDI MSI spectra of a characteristic mass signal at *3340* (*m/z*) was achieved using 15 layers of trypsin (*5 layers of trypsin* vs. *15 layers of trypsin 0.094 ± 0.003* vs. *0.356 ± 0.114* (*P < 0.05*); *10 layers of trypsin* vs. *15 layers of trypsin 0.131 ± 0.013* vs. *0.356 ± 0.114* (*n*.*s*.); *20 layers of trypsin* vs. *15 layers of trypsin 0.093 ± 0.003* vs. *0.356 ± 0.114* (*P < 0.05*); *n = 3*).

### Effect of the grid size for MALDI MSI analysis

Based on the grid size, the distance between subsequent MALDI MSI analysis positions was calculated. Thus, the grid size determines the pixel number and directly influences the resolution of the MALDI MSI data. To investigate the effect of grid size, size varied between 20 and 100 μm. Figure 3c shows characteristic MALDI MSI data using three grid sizes (20/50/100 μm). As expected, a decreased grid size resulted in a higher resolution of the resulting MALDI MSI data.

**Fig. 4:**
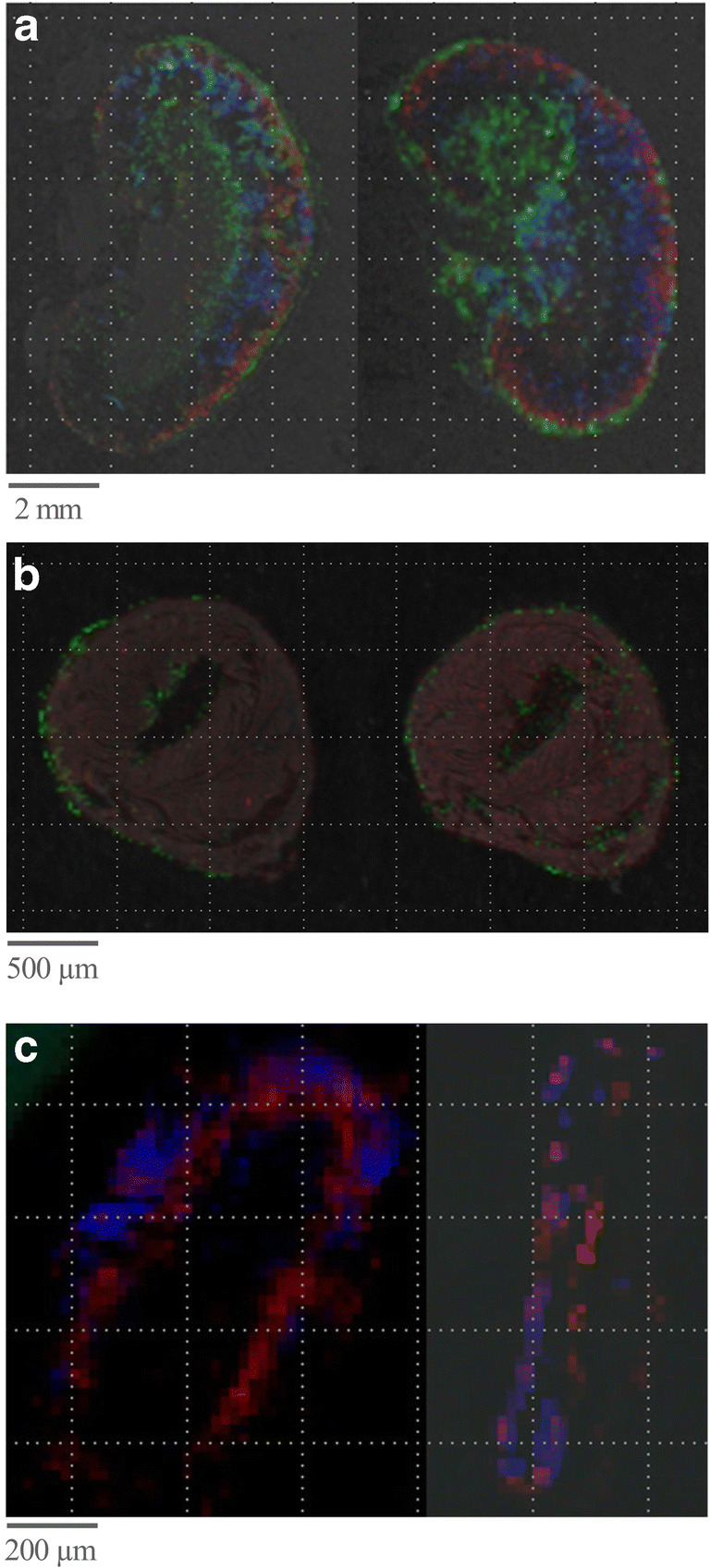
MALDI MSI images of FFPE tissues using the optimized SOP. Shown are MALDI MSI images of mouse organs using the optimized SOP and overlaid on the tissue scan. **a** Kidney, 184 (*m/z*) red, 828 (*m/z*) green, 932 (*m/z*) blue. *Grid size 50 μm. **b** Heart, 657 (*m/z*) green, 445 (*m/z*) red. *Grid size 50 μm. **c** Aorta, 864 (*m/z*) red and 237 (*m/z*) blue. *Grid size 20 μm

The reproducibility of the SOP and the application of the protocol independently from the tissue source were analyzed for validation of the optimized parameters. MALDI MSI data were accumulated from tissue sections of the mouse kidney, aorta, and heart (Fig. 4) using the following protocols based on the individual optimization steps above:

In addition to kidney tissue, tissue samples from the aorta and heart of mice were analyzed using the developed protocol to examine its applicability independently from the source of the tissue. Different mass signals were chosen to analyze specific regions of the tissue. Figure 4 shows the MALDI MSI image of the mouse kidney (Fig. 4a), heart (Fig. 4b), and aorta (Fig. 4c) acquired using this optimized SOP. The mass signals of 184 (*m/z*) red, 828 (*m/z*) green, and 932 (*m/z*) blue (Fig. 4a); 657 (*m/z*) green and 445 (*m/z*) red (Fig. 4b); and 864 (*m/z*) red and 237 (*m*/*z*) blue (Fig. 4c) are displayed. The results demonstrate the feasibility of this SOP to clearly distinguish and resolve individual anatomic features in each organ by imaging-selected molecular markers. For the kidney, a layered structure displaying the main, functionally different regions (the renal cortex, medulla, and pelvis) becomes clearly visible. Similarly, separate anatomic features are distinguishable in the aorta and heart. This demonstrates the applicability of the optimized SOP for the analyses of complex tissue samples from diverse tissue sources.

Furthermore, the interday variability of the total ion count of the whole spectra was quantified by analyzing mouse heart sections three times on three consecutive days using the optimized protocol. The results show no significant differences between the S/N ratios on consecutive days (analysis day 1 4.531*e^*9*^; analysis day 2 3.289*e^*9*^; analysis day 3 3.376*e^*9*^). The resulting interday variability of the S/N ratio was in the range of 15.16%. Table 1 presents an overview of the optimized steps of the SOP. The conditions given in italicized letters result in a maximized S/N ratio of the corresponding MS image.

### Applicability of the protocol for fresh-frozen tissue MALDI MSI analyses

**Fig. 5:**
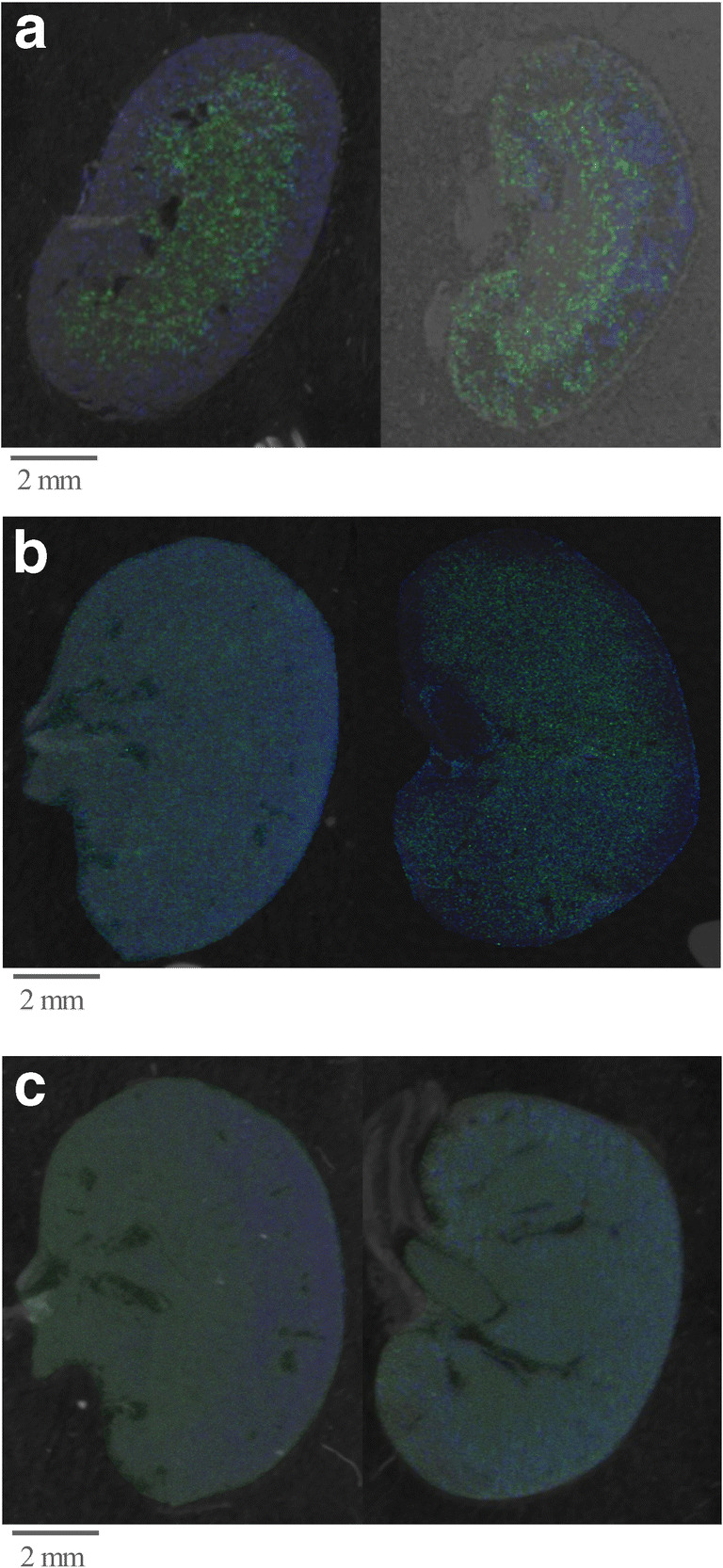
MALDI MSI images of fresh-frozen tissues (left image) compared with FFPE tissue samples (right) using the optimized SOP. Shown are MALDI MSI images of a mouse kidney using the optimized SOP and overlaid on the tissue scan. 718 (*m/z*) green, 958 (*m/z*) blue (**a**, **b** accumulated by using an Ultraflex mass spectrometer; **c** accumulated by using Rapiflex mass spectrometer (both from Bruker Daltonics, Bremen, Germany)). **a** Partial resolution 50 μm. **b** Partial resolution 25 μm. **c** Partial resolution 10 μm

Although this protocol was developed and optimized for the application of FFPE tissue samples, the applicability of this protocol for fresh-frozen tissue samples was tested as well. Figure 5a exemplary shows the MALDI MSI images of fresh-frozen tissues (Fig. 5a left) compared with an FFPE tissue sample (Fig. 5a right) from a mouse kidney. The mass signals 718 (*m/z*) green and 958 (*m/z*) blue are displayed. The results clearly demonstrate that the protocol is applicable for the analysis of fresh-frozen tissues. The improvements are of particular importance for high-resolution MSI spectra. Figure 5b shows the MALDI MSI images of fresh-frozen tissues (Fig. 5b left) compared with an FFPE tissue sample (Fig. 5b right) from mouse kidneys using a partial resolution of 25 μM using an Ultraflex as well as a partial resolution of 10 μM using a Rapiflex mass spectrometer (Fig. 5c left: fresh-frozen tissue sample; right: FFPE tissue sample) (both from Bruker Daltonics, Bremen, Germany).

## Discussion

With the advancement in the development of lasers and analyzers in the field of MALDI mass spectrometry, the research area of MALDI MSI has progressed accordingly. MALDI MSI has gained increasing impact on scientific studies and diagnostics [23, 24, 43].

Considering the progress in MALDI MSI technology in recent years, the question of further optimization of sample preparation emerges. Some protocols for sample preparation are available [15, 21, 29, 44], but these protocols were not systematically developed and/or optimized. Therefore, we have chosen a systematic approach within this study to develop, establish, and validate an optimized protocol for the sample preparation of FFPE tissue sections from the kidney, heart, or vessels. Initially, we focused on FFPE tissue sections, since these sections are readily available in the clinic and much easier to conserve than fresh-frozen tissue sections. However, the applicability of the SOP to frozen tissue sections was subsequently validated as well. To achieve an optimized sample preparation procedure, we systematically optimized each individual analytic step of the sample preparation (Table 1). Since different trypsin preparations might have an impact on the resulting mass spectra, the first step was to compare the effect of trypsin obtained from different manufacturers on the resulting MALDI MSI data. Our data confirm that the choice of trypsin preparation has a significant impact on the resulting MALDI MSI data, and regarding the resulting S/N ratio, the best result was achieved using trypsin purchased from Sigma-Aldrich.

In addition, the solvent conditions used to dissolve trypsin are highly relevant for the resulting S/N ratios of the MALDI MSI spectra. Hydroxymethyl-aminomethane hydrochloride and ammonium bicarbonate were compared as solvents for trypsin, as they are commonly used in enzymatic digestion processes. The most intense mass signals and S/N ratios of the resulting MALDI MSI data were obtained using trypsin dissolved in 20 mM ammonium bicarbonate. Concentrations above 20 mM of ammonium bicarbonate had a negative effect on the S/N ratio, most likely due to the negative effects on the ionization process as an essential step for MS data acquisition. Since tryptic enzymes are most active at physiological pH value near 7.4, we included 20 mM ammonium bicarbonate as the potential solvent for trypsin in our study. Although 20 mM ammonium bicarbonate provides the best MALDI MSI results, no significant differences in the resulting MALDI MSI images were detected.

After identification of the optimized solvent for trypsin for MALDI MSI, the concentration of trypsin was optimized to the maximal S/N ratio. While a low concentration of trypsin (5 nM) results in a low S/N ratio, most likely due to incomplete digestion of proteins, significantly increased S/N ratios were reached using a trypsin concentration of 50 nM. Next, the effect of different incubation conditions for trypsin digestion on the resulting MALDI MSI data was analyzed, in an attempt to obtain an efficient protein digestion (reflected by an increased number of mass signals): with the humid conditions in a closed chamber at a temperature of 37 °C, selected primarily for trypsin digestion, condensation of water on the tissue is formed. As a consequence, components of the tissue sections may diffuse out of the tissue and change their position. Optimized MALDI MSI data regarding the S/N ratio were reached without an additional incubation period for trypsin digestion, thus also avoiding potential incubation-associated inaccuracies in spatial information.

Since an additional delay during the spray process had no positive effects on the resulting S/N of the mass signals, a continuous spray process was chosen to expedite the process. Further, we analyzed the effect of air drying or vacuum drying of the tissue between the washing and trypsin coating steps using a vacuum desiccator vs. room temperature conditions. However, no significant differences were detected. Therefore, for practical purposes, drying at room temperature was chosen for preparation of FFPE tissue samples.

There are two common protocols for the tissue washing procedure for MALDI-MS application available in the recent literature [45, 46]. The “basic wash” uses 70%/100% isopropanol, whereas “Carnoy’s wash” additionally includes 0.1% TFA and “Carnoy’s solution” (ethanol-chloroform-acetic acid in 6:3:1 parts, respectively) for fixing. Both protocols (basic wash and Carnoy’s wash) include a washing step using alcoholic solutions. The washing process of the tissue sample is a critical step in sample preparation since during this process, components of the tissue section may be extracted. Therefore, the optimization of this preparatory step is highly relevant for sample preparation of tissue for MSI purposes. In our study, the use of Carnoy’s wash protocol before the antigen retrieval step resulted in an increased S/N ratio compared with the use of the described basic wash. This may be because Carnoy’s wash protocol included TFA, which enhances the ionization of the analytic ions, thus facilitating MALDI MSI measurements. Whether inclusion of an additional fixative in Carnoy’s wash (ethanol-chloroform-acetic acid fixation) also contributes to or influences the acquired MSI data was not separately examined in this study.

Next, we optimized the spray flow rate of trypsin coating as well as the number of coated trypsin layers using a MALDI matrix spotter. The highest signal intensities and S/N ratios were achieved using a spray flow rate of 5 μl/min and 15 trypsin layers, compared with 15 μl/min and fewer trypsin layers, respectively. Most likely, the increased amount of deposited trypsin per tissue area was associated with lower spray flow rates, and the increased number of trypsin layers resulted in a more complete digestion of proteins, thereby explaining the increased signal intensities and S/N ratios. This is also in accordance with the improved S/N ratio when using increased trypsin concentrations. Further, a lower spray flow rate may have the additional advantage of enabling a more homogeneous trypsin deposition. Although this has not been investigated in more detail in this study, this may have further positively impacted the reproducibility of our SOP. More than 15 layers did not further improve the S/N ratio and may have even further reduced the obtained S/N ratio: most likely an optimal protein digestion had already been reached and additional coating might merely interfere with subsequent ionization steps during MALDI MSI.

Finally, a decrease in the grid sizes caused a higher resolution of the resulting MS MSI spectra, as expected. Presently, the minimum grid sizes realizable by conventional MALDI MSI mass spectrometers are in the range of 20–30 μm. In the case of high-end MALDI MSI mass spectrometers like the Rapiflex mass spectrometer (Bruker Daltonics, Bremen, Germany), the minimum grid sizes are in the range up to 5–10 μm, which is still—at least in part—larger than the size of cellular compounds. It is expected that future developments in this field will further increase the resolution by decreasing grid sizes. Nonetheless, our study shows that even with the currently achievable grid sizes, MALDI MSI is already currently able to clearly resolve individual anatomic features in tissue samples of organs by imaging selected molecular markers using an optimized SOP. The detailed description of each individual optimization step ensures that the optimization algorithm is presented in a comprehensible way. Overall, the current protocol validates some preparation steps of protocols already described with the aim of developing a systematically optimized SOP. Therefore, some sample preparation steps of this SOP are comparable with other published protocols such as Casadonte et al. [30] or O’Rourke [31, 32].

For validation, and to examine the reproducibility of the tissue sample preparation, MALDI MSI measurements of a kidney section were repeated three times on consecutive days. No significant differences were observed between the S/N ratios, which demonstrates that the quality of the resulting MSI spectra is highly reproducible. Further, using our optimized SOP high-quality images of tissue sections of the kidney, heart, and aorta clearly resolves individual anatomic features within each organ based on selected molecular markers, which demonstrates the applicability of our SOP from varying tissue sources. Thus, this SOP is highly applicable for the analyses of FFPE tissue sections, validated as demonstrated for kidney, vessels, and heart tissues for MALDI MSI, focusing on analyses of peptides and proteins. In addition, this SOP is very likely suitable for the analyses of drugs and metabolites due to the comparable desorption characteristics in tissue samples as well. The treatment of tissue sections with solvents is problematic in that the spatial distribution of the tissue components is artificially altered. Since we analyzed consecutive tissue slides, and the quality of these tissue slides was histologically evaluated, the impact of this effect on the SOP development was greatly limited.

## Conclusion

The developed and validated SOP for MALDI MSI imaging of FFPE tissue sections will generate images with highest sensitivity, spatial resolution, and reproducibility. This will improve the comparability of data between different centres and thus contribute to the wider use of MALDI MSI in clinical routine as well as research studies. The step-by-step protocol can be found in the supplement.

## Abbreviations

FFPE: Formalin-fixed paraffin-embedded
HPLC: High-pressure liquid chromatography
ITO: Indium tin oxide
LC-MS: Liquid chromatography-mass spectrometry
MALDI MSI: Matrix-assisted laser desorption/ionization mass spectrometry imaging
RMS: Root mean square
S/N: Signal-to-noise ratio
TFA: Trifluoroacetic acid
